# The association between psychiatric diagnosis and violent re-offending in adult offenders in the community

**DOI:** 10.1186/1471-244X-8-92

**Published:** 2008-11-25

**Authors:** Martin Grann, John Danesh, Seena Fazel

**Affiliations:** 1 Department of Psychology, University of Stockholm, Sweden and Centre for Violence Prevention, Karolinska Institute, Stockholm, Sweden; 2 Department of Public Health and Primary Care, University of Cambridge, Cambridge, UK; 3 Department of Psychiatry, University of Oxford, Warneford Hospital, Oxford, UK; 4 Centre for Violence Prevention, Karolinska Institute, Stockholm, Sweden

## Abstract

**Background:**

High rates of repeat offending are common across nations that are socially and culturally different. Although psychiatric disorders are believed to be risk factors for violent reoffending, the available evidence is sparse and liable to bias.

**Method:**

We conducted a historical cohort study in Sweden of a selected sample of 4828 offenders given community sentences who were assessed by a psychiatrist during 1988–2001, and followed up for an average of 5 years for first violent offence, death, or emigration, using information from national registers. Hazard ratios for violent offending were calculated by Cox regression models.

**Results:**

Nearly a third of the sample (n = 1506 or 31.3%) offended violently during follow-up (mean duration: 4.8 years). After adjustment for socio-demographic and criminal history variables, substance use disorders (hazard ratio 1.97, 95% CI, 1.40–2.77) and personality disorders (hazard ratio 1.71, 1.20–2.44) were significantly associated with an increased risk of violent offending. No other diagnoses were related to recidivism risk. Adding information on diagnoses of substance use and personality disorders to data recorded on age, sex, and criminal history improved only minimally the prediction of violent offending.

**Conclusion:**

Diagnoses of substance use and personality disorders are associated with the risk of subsequent violent offending in community offenders about as strongly as are its better documented demographic and criminal history risk factors. Despite this, assessment of such disorders in addition to demographic and criminal history factors enhances only minimally the prediction of violent offending in the community.

## Background

High rates of repeat offending are common across nations that are socially and culturally different, including the US, UK, Australia, Germany, Netherlands, Iceland, Norway and Japan [1]. More than one in three offenders, whether given custodial or community sentences, are reconvicted within 2 years for a violent crime [2,3]. Risk assessment instruments, including the Violence Risk Appraisal Guide (VRAG) [4] and the Historical, Clinical and Risk management scheme (HCR-20) [5], are widely used in North America, the UK and other Western countries to help identify individuals at high risk of reconviction. Such instruments are typically completed by clinical interview and/or review of medical records, and involve scoring individuals on the basis of a set of socio-demographic, criminal history, and clinical risk factors based on research on repeat offending on leaving prisons and secure hospitals. These instruments can heavily influence decisions on sentencing of prisoners, the timing of their release, and the extent of supervision during probation and parole.

Although much is known about certain demographic risk factors for re-offending, which include young age, male sex, and a history of previous violent convictions [6], less is known about potentially modifiable psychiatric determinants. Previous research on mental health interventions to prevent violent repeat offending have been criticised for being methodologically flawed [7,8], and few have been done in non-custodial samples [9,10]. Nevertheless, most current risk assessment tools utilise information on the presence or absence of a diagnosis of major mental illnesses, personality disorder, and substance use disorders as factors to help stratify the risk of re-offending, despite the paucity of data on what extra information is actually provided by measurement of these factors [4,5].

One of the reasons for the uncertainty about the psychiatric determinants of offending is that their contribution is different depending on the sample being investigated. Four types of samples have been examined: general population, discharged psychiatric patients, mentally disordered offenders, and all offenders. In the general population, the psychoses have been demonstrated to be associated with increased risk of violent crime [11], even after adjustment for potential socio-demographic confounders and comorbid substance abuse [12,13]. In addition, there is evidence from general population samples that personality disorder [14] and substance use disorders [15,16] are associated with violent outcomes. One large study of discharged psychiatric patients has investigated risk factors for violence compared with a sample of community based individuals living in similar neighborhoods, and found that patient status did not increase the risk of violence but the presence of comorbid substance abuse in the patient sample did [17].

Evidence for risk factors for violent crime based on research in general population and discharged patient samples do not appear to be generalizable to offenders, where socio-demographic and criminal history factors are most strongly associated with repeat offending and in whom severe mental illness is in fact thought to be protective [6]. However, this research has mainly examined mentally disordered offenders rather than all offenders, and used within-group designs to compare risk factors. These studies have therefore little bearing on the issue of whether mental disorder *per se *is associated with repeat offending. In order to do so, it is necessary to have a comparison group of offenders whom have been assessed as not suffering from mental disorder.

We investigated 4828 offenders who were followed up for an average of about 5 years to quantify associations more reliably than before between diagnosis of psychiatric disorders and risk of re-offending. These offenders were selected on the basis of being court-referred for psychiatric assessment and given community (ie, non-custodial) sentences. The group comprised of offenders convicted of violent crime or non-violent crime, and some of those included for non-violent crimes had a prior history of violent crime. The current sample was selected for two main reasons. First, it involved a comparison group of offenders who were clinically assessed as having no psychiatric disorders, enabling us to assess the relative contribution of possible psychiatric diagnoses. Second, as it involved offenders given community sentences, its findings should be relevant to decisions about parole and probation.

## Method

In Swedish courts, as in most Western countries, psychiatric assessments can be ordered to inform decisions about sentencing and transfer to hospital. These assessments are routinely requested by the court in those with a known history of psychiatric disorder, or whose offence characteristics or behaviour in police custody has raised mental health concerns. These assessments are made by specialist (state-certified general adult psychiatrists with a specialty in forensic psychiatry) psychiatrists who review legal and medical records, and conduct a clinical interview in which standardised diagnoses are recorded according to the Diagnostic and Statistical Manual of Mental Disorders (DSM-III-R until 1996, and subsequently DSM-IV). Current diagnoses are made, and diagnostic instruments are not used. Diagnostic concordance of forensic psychiatry assessments in Sweden (in a different setting) with those made on discharge from the hospital register has been shown to be high for psychoses [18]. Schizophrenia diagnoses in the hospital register concord well with diagnoses obtained by an OPCRIT record review and interview (generating a DSM-IV diagnosis of schizophrenia) reflected in kappa values of 0.74–0.76 [19]. For substance use diagnoses, there is fair agreement (kappa = 0.32) between forensic psychiatry and hospital register diagnoses [18]. The crime register has total national coverage – only 0.05% of all registered convictions had incomplete personal identification numbers during 1988–2000 [11].

Information was retrieved on all 4828 court-ordered psychiatric assessments from 1993–2001 who received the following community-based sanctions: probation with or without community service (n = 2428 or 50.3%), conditional prison sentence (n = 338, 7.0%), or fine (n = 2062, 42.7%). In offenders with more than one court-ordered assessment, the first was used. Thus, offenders sentenced to prison, ordered to receive community treatment as part of their sentence, or transferred to hospital were excluded as the effect of psychiatric risk factors would be altered by medical and/or psychological interventions. Non-Swedish citizens who were deported as part of their sentence were also excluded, as were 10 individuals with missing information. This cohort of psychiatrically examined offenders represented 4.8% of all individuals who received community sentences over the same time period (n = 100,503), and the baseline characteristics of both groups were compared.

The date of the psychiatric assessment, typically a few weeks prior to sentencing, was used as the baseline. The retrospectively constructed cohort was then followed for reconvictions using the national crime register until either the date of first new violent offence (defined as homicide, assault, robbery, any sexual offence, arson, illegal threats and harassment), death, or end of follow-up (31 December 2001). The date of the first new violent offence was specifically the date of conviction. We chose conviction information, because, in Sweden, we would not miss those who were hospitalized to secure hospital – it is at sentencing, rather than conviction, that those with mental illness are hospitalized. In calculating time at risk, we did not take into account time spent in hospital as no assumption was made whether this would reduce or increase their risk of crime.

Ethics approval was received from Huddinge University Hospital, Stockholm (#194/02).

The following psychiatric diagnoses were investigated: organic disorders (diagnostic codes 290–4; n = 104), schizophrenia (diagnostic code 295; n = 248), bipolar disorder (diagnostic codes 296.0–.06 and 296.40–.89; n = 111), other psychoses (diagnostic code 297–298; n = 327), depression (diagnostic code 296.2–.36, 296.9 and 311; n = 308), anxiety (diagnostic code 300; n = 381), post-traumatic stress disorder and adjustment disorders (diagnostic codes 308–309; n = 122), any substance use disorder as principal or comorbid diagnosis (diagnostic codes 303–305; n = 2336), any personality disorder as principal or comorbid diagnosis (diagnostic code 301; n = 2159), mental retardation/learning disability (diagnostic codes 317–319; n = 138), and those with diagnoses that were deferred (diagnostic code 799.9; n = 791). Diagnoses were deferred where the examining psychiatrist was unable to make a diagnosis; such individuals were not included in the comparison group and did not contribute to the present analyses as no assumptions were made as to whether these individuals had a mental disorder or not.

Cox regression models were constructed for each diagnosis investigated. Hazard ratios were calculated using Cox regression, with progressive adjustment for age and sex, any previous violent conviction, type of index offence (violent *vs*. non-violent), and comorbid substance use disorders. To estimate the discriminative values of predictive models, we calculated the C-index and the area under the receiver-operating-characteristic-curve (AUC) in order to determine whether the sequential addition of psychiatric data improved the prediction beyond that of socio-demographic and criminal history factors on violent offending. The C-index is a discriminatory measure for the Cox regression model that is an extension to the AUC for the binary model. The C-index and AUC can be interpreted as the probability that, given two randomly selected participants, the participant who offends first has the worse prognosis, as predicted by the model. Being a probability, the C-index and AUC can range between 0 and 1. A value of 0.5 indicates no ability, beyond that of chance, of the model to discriminate between ranks of participants in terms of risk; a value of 1 indicates perfect discrimination; a value less than 0.5 would indicate discrimination in the opposite direction to that expected. The C-index includes comparisons between survival times and censoring times instead of simply using binary information, and hence is a measure in cohort studies preferred over AUC [20]. We have included AUC by way of comparison as this is the most widely used measure in the field [9].

Furthermore, in order to illustrate the predictive ability of the final model (which included all the significant risk factors from the Cox regression model), we calculated the positive predictive value – that is, the proportion of those predicted by the model to reoffend who actually reoffended, where a value of 0.5 corresponds to no predictive validity (chance) and a value of 1 suggests 100% accurate prediction. To do this, we had to assume a specificity or true negative rate, which we set at 0.9 (or 90%). The positive predictive value was then estimated by taking the cut-off score of the AUC of the final model where specificity was 0.90.

## Results

The total number of community-sentenced individuals included was 4828, with a mean age of 35.7 (*SD *= 11.5) at interview, of whom 419 (8.7%) were women, 1119 (23.2%) were non-Scandinavian citizens, and 1851 (38.3%) were convicted of a violent offence at baseline (the index offence). Follow-up time ranged from 4 to 2919 days (0–8 yrs) with a mean of 4.8 years (*SD *= 2.2). There were 243 (5.0%) offender deaths, at which point they were excluded from further follow-up. A total of 1506 (31.3%) individuals were reconvicted for violent crimes during follow-up.

The cohort was compared with all individuals who received community sentences during 1993–2001 (*N *= 100,503). Those who received community sentences without forensic psychiatric assessments were younger (*M *= 30.7, *SD *= 12.7) than the study cohort, and there were fewer non-Scandinavian citizens (5%). The proportion of women offenders and those with a violent index offence was similar (data not shown).

Crude hazard ratios for violent offending were significant for substance use disorders and personality disorders, and there were trends for an inverse relationship with schizophrenia and depression. After adjustment for age and sex, the hazard ratio for violent offending in individuals diagnosed with schizophrenia was 1.15 (95% CI, 0.73–1.81), which increased to 1.29 (0.82–2.03) after further adjustment for factors related to criminal history (Table 1). A similarly modest but non-significant trend was observed between a diagnosis of depression and risk of violent offending (Table 1). Other psychiatric diagnoses (including those that were deferred) were not significantly associated with violent offending (data available upon request). By contrast, the age and sex-adjusted hazard ratios in people diagnosed with substance use disorders and with personality disorders were 1.94 (1.38–2.72) and 1.89 (1.34–2.65), respectively, and remained highly significant (p < 0.001) and largely unchanged after further adjustment for criminal history factors (Table 1). Table 2 shows that C-index for the occurrence of violent offending was 0.60 after inclusion of data on age and sex, 0.62 after addition of further data on criminal history, and, 0.63 after addition of further data on diagnosis of substance use disorder and personality disorder. The AUCs were 0.57 (95% CI: 0.55–0.59) after inclusion of data on age and sex, 0.62 (0.61–0.64) after addition of further data on criminal history, and, 0.65 (0.63–0.67) after addition of further data on diagnosis of substance use disorder and personality disorder.

**Table 1:** Hazard ratios of psychiatric risk factors for repeat violent offending in a cohort of community based offenders.

			**Hazard ratio (95% CI) adjusted cumulatively for**			
			
**Model**	**Number of violent re-offenders (%)**	**Number of non-re-offenders (%)**	**Age and sex**	**+ Any previous violent conviction**	**+ Index violent offence**	**+ Comorbid substance use disorder**
No psychiatric disorder	35 (22%)	124 (78%)	1.00	1.00	1.00	1.00
I. Schizophrenia	58 (23%)	190 (77%)	1.15 (0.73–1.81)	1.21 (0.77–1.89)	1.29 (0.82–2.03)	1.28 (0.77–2.11)
II. Depression	69 (22%)	239 (78%)	1.28 (0.83–1.96)	1.31 (0.86–2.00)	1.31 (0.86–2.00)	1.28 (0.82–2.00)
III. Substance use disorders as principal or comorbid diagnosis	848 (36%)	1488 (64%)	1.94 (1.38–2.72)	1.82 (1.30–2.56)	1.97 (1.40–2.77)	NA
IV. Personality disorder as principal or comorbid diagnosis	753 (35%)	1406 (65%)	1.89 (1.34–2.65)	1.80 (1.28–2.53)	1.91 (1.36–2.68)	1.71 (1.20–2.44)

Note: Cox regression models with time to violent re-offending as the dependent variable (re-offending base rate 31%) in psychiatrically assessed offenders followed during probation for an average of 4.8 years. Each diagnostic category is compared with the group of 159 offenders (re-offending base rate 22%) who had no psychiatric diagnosis.

**Table 2:** Hazard ratios and the incremental predictive value of socio-demographic, criminal history, and psychiatric risk factors for violent re-offending.

**Risk factors with hazard ratios (95% CI)**	**C-index scores**	**AUC (95% CI) **
Socio-demographic factors:		
Age, 1.08 (1.05 – 1.10) per 5 year decrease		
Male sex, 2.02 (1.59 – 2.56)	0.60	0.57 (0.55–0.59)
		
Criminal history variables*:		
Any previous violent conviction, 2.94 (2.37 – 3.65)		0.62 (0.61–0.64)^+^
Index violent offence, 1.25 (1.13 – 1.39)	0.62^+^	
		
Psychiatric variables*:		
Any substance use disorder, 1.94 (1.38 – 2.72)		
Any personality disorder, 1.89 (1.34 – 2.65)	0.63^++^	0.65 (0.63–0.67)

* adjusted for age and sex

+ Predictive validity of socio-demographic and criminal history variables

++ Predictive validity of socio-demographic, criminal history and psychiatric variables

Note: Left panel are hazard ratios for violent re-offending derived from Cox regression.

Middle panel are C-index scores of the additive predictive value of each factor derived from regression coefficients from Cox regression models, and right-hand panel are the corresponding AUC values included for comparison.

In addition, we ran separate analyses of men and women. The hazard ratios did not change significantly for the men, but the data was underpowered to examine women separately. We also ran the analyses by three agebands (under 25, 25–39, 40+ years) and also did not find significant differences (data not shown).

Assuming a specificity of 90%, the positive predictive value was approximately 40% – i.e., 40% of offenders who were predicted by the full model (that included data on demographic, forensic and diagnostic factors) to reoffend violently did actually do so during the follow-up period.

## Discussion

In this study, 4828 community offenders were followed for an average of about 5 years to help quantify associations between psychiatric disorders and risk of violent offending. Adjusted hazard ratios for violent offending were approximately two-fold in individuals diagnosed with either substance use disorders or personality disorders, comparable in size to associations observed in these same individuals with demographic and criminal history risk factors. Even so, diagnostic information on these psychiatric disorders provided minimal additional predictive value beyond that provided by age, sex and criminal history. Diagnoses of schizophrenia or depression were weaker correlates of violent conviction and were not statistically significant in the current study; larger numbers will be needed to assess any modest effects of these diagnoses more precisely.

Our study is in keeping with recent work on mentally disordered offenders discharged from secure hospitals that has confirmed the importance of static factors, such as age and gender for predicting re-offending [21,22]. Furthermore, one study has shown increased risk of re-offending for individuals with personality disorders [22], which was also demonstrated in the current report. In contrast with that study, we found that substance use disorders were associated with a modest risk increase. However, our data are not directly comparable as we have used a non-mentally disordered comparison group and most patients in secure hospitals have primary diagnoses of schizophrenia. Despite these differences, it is notable that our finding of the limited value in adding psychiatric diagnostic information to risk prediction has also been found in patients discharged from one high security hospital in England [21].

It may be counter-intuitive to conclude that disorders that nearly doubled the chance of violent reoffending can add only minimally to prediction of violent reoffending. However, a factor that increases risk does not necessarily mean that knowledge of that factor can be readily used to predict an event before it occurs. This is illustrated by the modest C-Index and AUC scores reported in this study (Table 2), and the minimal improvements seen when adding diagnostic information into the model which already includes the major established risk factor.

As many countries are enacting or contemplating legislation that curtails freedom of people with mental illnesses in the pursuit of community safety, the present data underscore the need for additional research on the topic to ensure that public policy is evidence-based [7]. Limitations of the present data highlight the need for such work. Although this study involved community-based sanctions (thereby avoiding problems inherent in prison-based or secure hospital studies, where possible risk factors are likely to be altered by the experience of institutionalisation and interventions received), it may mainly reflect the less severe spectrum of offending. It may also reduce the strength of any association with mental illness as those whose offences were thought to be related to their psychiatric condition may have been more likely to be given a custodial sentence or admitted to secure hospital. As participants in the current study were selected on the basis of court referral for psychiatric examination, they were somewhat different from the larger pool of Swedish citizens given community sentences (e.g. they were older and less likely to be Scandinavian). It should be made clear that the study did not assess risk for violent reoffending associated with a diagnosis for all of the offenders with noncustodial sentences but only a small proportion, namely, those referred for court-ordered forensic psychiatric examination. Given the likely reasons for ordering an assessment, the cases that were assessed, but did not receive a diagnosis, may have been different from those not assessed (e.g., more likely to have subclinical levels of symptoms). Further work is needed, therefore, to determine to what extent the current findings can be extrapolated to other settings. This would be helpful to identify whether the comparison group used in the study was the most appropriate – those who were referred for psychiatric evaluation but were assessed as having no mental disorder – who could, for example, be compared with those with no history of mental health problems according to their records. The present study did not address clinical factors beyond psychiatric diagnoses, such as compliance with medication [23], social support, service provision, specific symptoms [24], different types of substance abuse (including alcohol compared with other illegal drugs), subthreshold substance abuse problems (such as binge drinking) and personality traits [25] which may be of particular relevance to offenders with mental illnesses. The lack of information on service provision for the sample and engagement with these services is another limitation, and future research could explore its potential impact. Diagnoses in this report were made by specialist psychiatrists using internationally-based standardised criteria, and the validity of forensic assessments is reported to be moderate to high compared with inpatient diagnostic assessments [18], but this meant that information on personality traits was lacking and no information on inter-rater reliability was available. The present report is based on routinely collected data, and although using structured instruments would improve the diagnostic validity of the investigation, this would likely limit the numbers that could be recruited in any such follow-up study.

Another limitation with routinely collected information on offending is that it underestimates the true prevalence of violence and antisocial behavior [17]. However, based on the findings of other research, this underestimate of true violent offending is unlikely to affect the accuracy of risk ratios. Evidence from the Dunedin cohort that was followed up for violent crime found that the extent of the underestimation of violence was similar in mentally disordered individuals and controls [26]. Furthermore, using conviction data has the advantage of avoiding the reporting biases associated with self-report and informant questionnaires for crime and potentially allows for international comparisons.

Risk assessment instruments are widely used by mental health and criminal justice systems to help identify high-risk offenders and estimate the likelihood of re-offending. Although the evidence about socio-demographic and criminal history factors for repeat offending is substantial, comparatively little is known about psychiatric risk factors, particularly in the community setting. The present study advances knowledge on psychiatric risk factors in violent re-offending because it involves large numbers, prolonged follow up, a community-based sample, robust ascertainment of psychiatric disorders, and it is the first, to our knowledge, to include a comparison group with no psychiatric disorder at baseline. Notwithstanding the fact that the sample is selected, it suggests two main conclusions. First, there may be scope for the prevention of violent offending in the community by improved treatment and management of common psychiatric disorders, particularly of substance use and personality disorders, but further work is needed to help assess whether these associations are causal and reversible. Second, the present data suggest that incorporating information on psychiatric diagnoses in forensic risk assessments probably yields little incremental predictive gain – at least in the community.

## Conclusion

In summary, in this selected sample of community offenders, we found that diagnoses of substance abuse and personality disorders are risk factors for violent offending comparable in magnitude to established socio-demographic factors, but that their assessment provides minimal incremental predictive value.

## Competing interests

The authors declare that they have no competing interests.

## Authors' contributions

MG, JD and SF designed the study. MG and SF analysed the data, and with JD interpreted the results. All authors read and approved the final manuscript.

## Pre-publication history

The pre-publication history for this paper can be accessed here:


